# SUMOylation in α-Synuclein Homeostasis and Pathology

**DOI:** 10.3389/fnagi.2020.00167

**Published:** 2020-06-25

**Authors:** Mor Savyon, Simone Engelender

**Affiliations:** Department of Biochemistry, The B. Rappaport Faculty of Medicine and Institute of Medical Research, Technion – Israel Institute of Technology, Haifa, Israel

**Keywords:** Parkinson’s disease, α-synuclein, post-translational modifications, SUMOylation, ubiquitination, protein aggregation

## Abstract

The accumulation and aggregation of α-synuclein are central to Parkinson’s disease (PD), yet the molecular mechanisms responsible for these events are not fully understood. Post-translational modifications of α-synuclein regulate several of its properties, including degradation, interaction with proteins and membranes, aggregation and toxicity. SUMOylation is a post-translational modification involved in various nuclear and extranuclear processes, such as subcellular protein targeting, mitochondrial fission and synaptic plasticity. Protein SUMOylation increases in response to several stressful situations, from viral infections to trauma. In this framework, an increasing amount of evidence has implicated SUMOylation in several neurodegenerative diseases, including PD. This review will discuss recent findings in the role of SUMOylation as a regulator of α-synuclein accumulation, aggregation and toxicity, and its possible implication in neurodegeneration that underlies PD.

## Introduction

Parkinson’s disease (PD) is an incurable neurological disorder that affects over six million individuals worldwide and is predicted to increase as the population ages (Dorsey et al., 2018). Brains of PD patients exhibit extensive loss of dopaminergic neurons, responsible for the cardinal motor symptoms of the disease- resting tremor, bradykinesia, muscle rigidity and postural instability. Dysfunction of lower brain regions and peripheral nervous system underlies the non-motor features of the disease, including constipation, sleep disorders, and depression (Engelender and Isacson, 2017). Despite the availability of symptomatic treatments, there is still no intervention that halts the progression of the disease (Oertel and Schulz, 2016).

While the molecular mechanisms involved in the neuronal loss in PD remain unknown, the misfolding and accumulation of α-synuclein in PD brains seem to play a pathological role in the disease. In accordance, brains of patients with sporadic and familial forms of the disease contain proteinaceous inclusion bodies, known as Lewy bodies, which are predominantly composed of aggregated α-synuclein (Spillantini et al., 1997, 1998). Nevertheless, although Lewy bodies are a pathological hallmark of the disease, its role in neuronal toxicity remains controversial. Several studies have shown that oligomeric forms of α-synuclein are toxic to neurons (Outeiro et al., 2008; Karpinar et al., 2009; Kalia et al., 2013), and *in vitro* studies found that oligomerization and not fibrillation is common to different α-synuclein disease mutations (Conway et al., 2000). Furthermore, *in vivo* studies reported that mice expressing oligomer-prone α-synuclein mutants have a more severe dopaminergic loss compared to those expressing α-synuclein mutants that are more prone to form fibrils (Winner et al., 2011).

On the other hand, several studies pointed at the possibility that more organized α-synuclein fibrils, similar to those found in Lewy bodies, may also be toxic (Alam et al., 2019). In agreement, injection of pre-formed α-synuclein fibrils to brains is toxic to neurons promoting aggregation of endogenous α-synuclein and neurodegeneration (Luk et al., 2012). Based on these studies, both oligomeric and fibrillar α-synuclein species may contribute to neuronal toxicity. Regardless of the exact α-synuclein species that are more prone to cause toxicity, understanding how it accumulates in PD is essential to disclose the mechanisms that lead to disease.

Exposure of different cell lines to stressors, such as oxidative stress (Ostrerova-Golts et al., 2000), proteotoxic stress (Tanaka et al., 2001), nitrosative stress (Paxinou et al., 2001) and improper calcium homeostasis (Hettiarachchi et al., 2009), promote the accumulation and aggregation of α-synuclein. Exposure to stressors change post-translational modifications (PTMs) of proteins (Appella and Anderson, 2001; Kramer et al., 2015); therefore, it is conceivable that different types of cellular stresses may change α-synuclein PTMs, including phosphorylation (Fujiwara et al., 2002), truncation (Baba et al., 1998), nitration (Paxinou et al., 2001), glycation (Vicente Miranda et al., 2017), ubiquitination (Hasegawa et al., 2002), SUMOylation (Dorval and Fraser, 2006; Rott et al., 2017) and acetylation (Anderson et al., 2006). Thus, changes in α-synuclein PTMs may represent a missing link in the pathogenesis of PD.

## The Biology of α-Synuclein

α-synuclein is a 140 amino acid protein predominantly expressed in neurons. It is located at the presynaptic nerve terminals in close association with synaptic vesicles (Maroteaux et al., 1988; Withers et al., 1997; Kahle et al., 2000; Ribeiro et al., 2002). α-synuclein is an intrinsically disordered protein that lacks a stable three-dimensional structure (Weinreb et al., 1996; Conway et al., 1998; Davidson et al., 1998). Still, it is divided into three main domains: i) an N-terminal region (amino acids 1-60) containing several conserved KTKEGV repeats that render a more alpha-helical structure and enables interaction with membranes (Davidson et al., 1998; Jo et al., 2000; McLean et al., 2000); ii) a central portion containing a hydrophobic amino acid stretch known as the non-amyloid component of the amyloid plaques (NAC; amino acids 61-95) and that is involved in the formation of beta-sheet structure and aggregation (Bodles et al., 2001; Murray et al., 2003); and iii) a C-terminal region (amino acids 96-140), which is abundant in negatively charged amino acid residues (Park et al., 2002; Murray et al., 2003). Despite its relatively disordered structure, α-synuclein can shift into a more compact conformation, which conceals the NAC region and prevents its exposure to the cytoplasm, withholding its tendency to aggregate spontaneously (Theillet et al., 2016). In agreement with this possibility, the cleavage of α-synuclein C-terminal region promotes its aggregation and toxicity in cell lines and mice (Tofaris et al., 2006), suggesting that the C-terminal region may play a critical role in concealing α-synuclein NAC region.

The exact function of α-synuclein is still unclear. Nevertheless, studies have connected it with the regulation of synaptic vesicle pool (Murphy et al., 2000; Cabin et al., 2002), the release of neurotransmitters (Abeliovich et al., 2000), SNARE-complex assembly (Burre et al., 2010) and dilation of exocytotic fusion pore (Logan et al., 2017). α-synuclein was also reported to interact with additional sub-cellular organelles and compartments, including the mitochondria (Devi et al., 2008) and the nucleus (Goers et al., 2003; Pinho et al., 2019).

Mutations (A53T/E, A30P, E46K, H50Q, and G51D) and multiplications of the α-synuclein gene (SNCA) cause autosomal dominant PD (Polymeropoulos et al., 1997; Kruger et al., 1998; Singleton et al., 2003; Chartier-Harlin et al., 2004; Zarranz et al., 2004; Appel-Cresswell et al., 2013; Lesage et al., 2013; Pasanen et al., 2014), yet the exact contribution of each mutation to the disease is not clear. Nevertheless, since the known mutations are located in the N-terminal region of the protein, changes in α-synuclein binding with membranes could play a role in the neuronal dysfunction caused by the PD mutants (Davidson et al., 1998; McLean et al., 2000). Another possibility, perhaps not mutually exclusive, is that increased oligomerization and aggregation (but not fibrillation) may be responsible for the toxicity of α-synuclein PD mutants (Conway et al., 2000). Supporting this idea, families with SNCA multiplications have increased levels of α-synuclein, increased α-synuclein aggregation and prominent neurodegeneration (Singleton et al., 2003; Book et al., 2018).

## Overview of α-Synuclein Post-Translational Modifications

Post-translational modifications are major cellular events that occur in response to endogenous and exogenous stimuli, allowing the cell to function in normal and stressful conditions. The development of mass spectrometry technology allowed gathering information regarding PTMs and how they regulate protein activity, localization, and stability (Nørregaard Jensen, 2004; Ren et al., 2014).

Numerous α-synuclein PTMs have been identified over the years, including phosphorylation, ubiquitination, truncation, nitration, glycation, acetylation, and SUMOylation. The most comprehensively studied PTM is phosphorylation. α-synuclein is extensively phosphorylated at Ser129, as identified by mass spectrometry in purified Lewy bodies (Fujiwara et al., 2002; Anderson et al., 2006). Different protein kinases were shown to phosphorylate α-synuclein at Ser129, including casein kinases, G protein-coupled receptor kinases and polo-like kinases (Okochi et al., 2000; Pronin et al., 2000; Mbefo et al., 2010). Accordingly, phosphorylation at Ser129 leads to increased α-synuclein fibrillation *in vitro* (Fujiwara et al., 2002) as well as aggregation and toxicity in different cell lines and transgenic models (Kahle et al., 2002; Smith et al., 2005; Chau et al., 2009; Chen et al., 2009; Zhou et al., 2011) (for more details see Table 1). Moreover, phosphorylation at Ser129 also changes α-synuclein localization and properties. For instance, it was shown to increase the nuclear localization of α-synuclein in H4 cells (Pinho et al., 2019), to modulate the binding of α-synuclein to membranes (Visanji et al., 2011; Kuwahara et al., 2012) and to increase its binding affinity to metal-ions (Lu et al., 2011), suggesting that phosphorylation at Ser129 may regulate both α-synuclein normal and pathological functions.

**TABLE 1:** α-synuclein post-translational modifications.

PTM	Site	Method of identification in α-synucleinopathy tissues	Putative functions
Phosphorylation	Ser129	Mass spectrometry of purified LBs (Anderson et al., 2006)/insoluble fractions from DLB brains (Fujiwara et al., 2002)	Increases α-synuclein insolubility in transgenic mouse oligodendrocytes and PD brains (Kahle et al., 2002; Zhou et al., 2011)
			Increases α-synuclein oligomerization in flies (Chen et al., 2009)
			Increases the formation of α-synuclein cytoplasmatic inclusion in SH-SY5Y cells (Smith et al., 2005)
			Increases α-synuclein neurotoxicity in SH-SY5Y cells and flies (Chau et al., 2009; Chen et al., 2009)
			Co-localizes with monoubiquitinated α-synuclein in α-synucleinopathy brains (Hasegawa et al., 2002)
			Increases nuclear accumulation of α-synuclein in H4 cells (Pinho et al., 2019)
			Modulates the binding of α-synuclein to membranes in SH-SY5Y cells and mouse neural precursor cells (Visanji et al., 2011; Kuwahara et al., 2012)
			Increased α-synuclein fibrillation *in vitro* (Fujiwara et al., 2002)
			Increased binding affinity to metal-ions (Lu et al., 2011)
	
	Ser87	Western blot analysis of and immunohistochemistry of α-synucleinopathy brains using anti-phospho-Ser87 α-synuclein antibodies (Paleologou et al., 2010)	Inhibits α-synuclein fibrillation and binding to membranes *in vitro* (Paleologou et al., 2010) Reduces α-synuclein aggregation and toxicity in a PD rat model (Oueslati et al., 2012)
	
	Tyr125	Western blot analysis of DLB brain lysates using anti-phospho-Tyr125 α-synuclein antibodies (Chen et al., 2009)	Reduces α-synuclein oligomerization and neurotoxicity in flies (Chen et al., 2009) Reduces oligomerization *in vitro* (Negro et al., 2002)
	
	Tyr39	Western blot analysis and immunohistochemistry of PD brains using anti-phospho-Tyr39 α-synuclein antibodies (Mahul-Mellier et al., 2014; Brahmachari et al., 2016)	Enhances α-synuclein aggregation in HEK293 cells (Brahmachari et al., 2016) Prevents α-synuclein degradation via the autophagy and proteasome pathways in primary cortical neurons (Mahul-Mellier et al., 2014)

Nitration	Tyr39, Tyr125, Tyr133, Tyr136	Immunohistochemistry of α-synucleinopathy brains using antibodies specific to nitrated α-synuclein residues (Giasson et al., 2000)	Increases α-synuclein aggregation in HEK293 cells (Paxinou et al., 2001) Reduces the affinity of α-synuclein to synthetic vesicles and accelerated the rate of fibril formation by unmodified α-synuclein (Hodara et al., 2004)

Truncation	C-terminal truncations Asn103 Asp115, Asp119, Asn122, Tyr133, Asp135 Glu139	Mass spectrometry of purified LBs (Anderson et al., 2006)/soluble and insoluble fractions from PD and DLB brains (Ohrfelt et al., 2011; Kellie et al., 2014; Bhattacharjee et al., 2019)	Increases α-synuclein inclusion formation and promotes neurodegeneration in α-synuclein (1-120) transgenic mice and flies (Tofaris et al., 2006; Periquet et al., 2007) Assembly of C-terminally truncated α-synuclein into disease-like filaments *in vitro* (Crowther et al., 1998) Induce rapid co-aggregation with full-length α-synuclein *in vitro* and SH-SY5Y cells (Liu et al., 2005)
	
	N-terminal truncations 5,39,65, 66,68,71–140	Mass spectrometry of soluble and insoluble fractions from PD brains (Kellie et al., 2014)	Deletion of first 10 or 30 amino acids change the structure of α-synuclein fibrils *in vitro*, reducing their stability and prompting increased cross-seeding and α-synuclein pathology in wild-type mice (Terada et al., 2018)

Glycation		Western blot analysis of thermo-enriched α-synuclein from PD and DLB brains using an antibody against advanced glycation end products (anti-CEL) (Vicente Miranda et al., 2017)	Increased α-synuclein aggregation and toxicity in yeast and H4 cells, dopaminergic LUHMES cells and α-synuclein transgenic mice (Vicente Miranda et al., 2017) Promotes *in vitro* oligomerization and impairs binding to lipid vesicles (Vicente Miranda et al., 2017) Increases α-synuclein aggregation *in vitro* and lead to toxicity when applied to SH-SY5Y cells (Chen et al., 2010)

Acetylation	N-terminal	Mass spectrometry of purified LBs (Anderson et al., 2006)/soluble and insoluble fractions from PD and DLB brains (Ohrfelt et al., 2011; Kellie et al., 2014; Bhattacharjee et al., 2019)	Prevents α-synuclein aggregation and toxicity in primary neurons and mice (de Oliveira et al., 2017)

Ubiquitination	Lys12 Lys21 Lys23	Mass spectrometry of purified LBs from DLB brains (Anderson et al., 2006)	Mono- and polyubiquitination promotes α-synuclein proteasomal degradation (Shin et al., 2005; Rott et al., 2011) Ubiquitination with K63-linked chains leads to α-synuclein (endo)lysosomal degradation (Shin et al., 2005; Tofaris et al., 2011) Accumulation of ubiquitinated α-synuclein results in increased aggregation and toxicity (Rott et al., 2008, 2011)

SUMOylation		Western blot analysis of immunoprecipitated α-synuclein from PD brain lysates (Rott et al., 2011)	SUMOylation by SUMO1 increases α-synuclein aggregation in SH-SY5Y (Rott et al., 2017) and COS-7 cells (Oh et al., 2011) Preventing SUMOylation increases α-synuclein aggregation and toxicity in HEK293 cells and a PD rat model (Krumova et al., 2011) Artificially SUMOylated α-synuclein decreases fibrillation *in vitro* (Krumova et al., 2011; Abeywardana and Pratt, 2015) (See more details in Table 2)

α-synuclein is also phosphorylated at Ser87, which was first detected in HEK293 and PC12 cells by phosphoamino-acid analysis and site-directed mutations (Okochi et al., 2000). Its relevance to the disease was confirmed by immunostaining of PD tissues using an anti-phospho-Ser87 α-synuclein antibody (Paleologou et al., 2010). In contrast to Ser129, phosphorylation at Ser87 inhibits α-synuclein fibrillation and binding to membranes *in vitro* (Paleologou et al., 2010), and also reduces α-synuclein aggregation and toxicity in a PD rat model (Oueslati et al., 2012; Table 1).

Phosphorylation of α-synuclein also occurs at tyrosine residues. Mutation analysis studies revealed that tyrosine kinases, such as Fyn and Syk, phosphorylate α-synuclein at tyrosine residues 125, 133 and 136 (Nakamura et al., 2001; Negro et al., 2002). Among these sites, phosphorylation at Tyr125 was detected in brain lysates by Western blot analysis using antibodies against phospho-Tyr125 α-synuclein and was found to be reduced in patients with Dementia with Lewy bodies (DLB) (Chen et al., 2009; Table 1), suggesting that the increased P-Ser129/P-Tyr125 ratio could contribute to the pathogenicity of α-synuclein.

More recently, the tyrosine kinase c-Abl was shown to interact with and to phosphorylate α-synuclein at tyrosine residue 39 (Mahul-Mellier et al., 2014; Brahmachari et al., 2016). Using antibodies against phospho-Tyr39 α-synuclein, this phosphorylation was found to be increased in lysates from substantia nigra of PD brains and to accumulate in Lewy bodies (Brahmachari et al., 2016; Table 1). Different from Tyr125, phosphorylation at Tyr39 enhances α-synuclein aggregation in HEK293 cells (Brahmachari et al., 2016), and prevents the proteasomal and autophagic degradation of α-synuclein in primary neurons (Mahul-Mellier et al., 2014). These findings are promising as a c-Abl inhibitor that crosses the blood-brain barrier, nilotinib, promotes α-synuclein autophagic degradation, and prevents dopaminergic degeneration in PD mouse models overexpressing α-synuclein (Hebron et al., 2013). Nevertheless, nilotinib failed to prevent α-synuclein aggregation and neuronal loss in a mouse model of MSA (Lopez-Cuina et al., 2020), suggesting that c-Abl inhibitors may be more effective to treat α-synucleinopathies with predominant α-synuclein neuronal accumulation.

Acetylation is also an outstanding α-synuclein PTM as it is found in Lewy bodies and brain regions affected in the disease (Anderson et al., 2006; Ohrfelt et al., 2011; Kellie et al., 2014; Bhattacharjee et al., 2019). Obliteration of α-synuclein acetylation sites *in vivo* leads to its enhanced aggregation and toxicity in primary neurons and mice (de Oliveira et al., 2017), implying that acetylation may prevent α-synuclein aggregation. Other PTMs, such as nitration and glycation, which are respectively related to nitro-oxidative stress and increased levels of blood glucose, are, on the other hand, associated with increased aggregation and toxicity of α-synuclein (Giasson et al., 2000; Paxinou et al., 2001; Vicente Miranda et al., 2017) (for more details see Table 1). Thus, the roles of PTMs are diverse, and their combined contribution to the pathogenesis of the disease is still far to be understood.

α-synuclein is also conjugated to polypeptides, such as ubiquitin and SUMO (small ubiquitin-related modifier). Biochemical separation and mass spectrometry analysis of purified Lewy bodies consistently identified the conjugation of α-synuclein by one, two, or three ubiquitin molecules, specifically at lysines 12, 21 and 23 (Hasegawa et al., 2002; Tofaris et al., 2003; Anderson et al., 2006). α-synuclein is degraded by the proteasome in cell lines and mice brain tissues (Bennett et al., 1999; Webb et al., 2003; Ebrahimi-Fakhari et al., 2011; Rott et al., 2011). Furthermore, m*ono-* and *polyubiquitination* by the E3 ubiquitin-ligases SIAH and CHIP promote the proteasomal degradation of α-synuclein (Shin et al., 2005; Rott et al., 2011; Table 1). In contrast, conjugation of ubiquitin chains formed at lysine 63 by the E3 ubiquitin-ligase Nedd4 leads to endo-lysosomal degradation (Tofaris et al., 2011). Most importantly, impairment of degradation pathways leads to the accumulation of ubiquitinated α-synuclein, rendering increased α-synuclein aggregation and toxicity in cell lines (Rott et al., 2008, 2011).

SUMOylation is another type of polypeptide conjugation that is essential for nuclear and extranuclear functions (Henley et al., 2018) and is responsive to cellular stressors (Enserink, 2015). SUMOylation also covalently modifies α-synuclein. We argue in this review that increased levels of SUMOylation may compete with ubiquitin and prevent α-synuclein degradation (Rott et al., 2017; Rousseaux et al., 2018). Further details on the different roles of SUMOylation on α-synuclein functions will be discussed below in detail.

Truncation of α-synuclein was initially described by biochemical means in purified Lewy bodies (Baba et al., 1998). Later, it was identified by mass spectrometry analysis in both purified Lewy bodies (Anderson et al., 2006; Bhattacharjee et al., 2019) and lysates of PD tissues (Ohrfelt et al., 2011; Kellie et al., 2014), and shown to occur at different α-synuclein residues at the C- and N-termini (for more details see Table 1). Although not all reported α-synuclein truncations were found enriched in Lewy bodies or PD tissues (Ohrfelt et al., 2011; Kellie et al., 2014; Bhattacharjee et al., 2019), the importance of truncations is implied by their increased tendency to aggregate *in vitro*, in cell lines and *in vivo* models (Crowther et al., 1998; Liu et al., 2005; Tofaris et al., 2006; Periquet et al., 2007). Also, the C-terminally truncated α-synuclein species assemble into filaments that resemble those found in diseased brains (Crowther et al., 1998) and leads to the formation of pathological inclusions and neurotoxicity in transgenic mice (Tofaris et al., 2006).

Although the development of protocols for Lewy body purification and solubilization from brain tissues has allowed an in-depth characterization of α-synuclein PTMs in PD and other α-synucleinopathies (Iwatsubo et al., 1996; Anderson et al., 2006), some PTMs, such as SUMOylation, nitration and glycation, have not been detected in α-synucleinopathy brains using conventional mass spectrometry techniques (Anderson et al., 2006; Bhattacharjee et al., 2019). Nevertheless, they have been identified in Lewy bodies of PD and DLB tissues by immunohistochemistry, and confirmed by biochemical means to directly modify α-synuclein (Giasson et al., 2000; Danielson et al., 2009; Kim et al., 2011; Rott et al., 2017; Vicente Miranda et al., 2017). The reason for failing to identify these PTMs by mass spectrometry in α-synucleinopathy brains is still not clear. One possibility is that some PTMs are more labile than others and are more easily deconjugated before analysis (Dapic et al., 2019). It is also possible that solubilization may not be complete before mass spectrometry analysis. For instance, even when brain lysates were incubated with stringent buffers containing detergents, such as sarkosyl, insoluble material from Lewy bodies was not entirely solubilized (Iwatsubo et al., 1996). Besides, technical limitations in the mass spectrometry technique *per se* could be responsible for this constraint. For instance, most protocols employed in traditional mass spectrometry analysis use proteolytic digestion before the electrospray ionization, rendering fragments that can be challenging in terms of size and charge to be detected by the mass spectrometer equipment (Dapic et al., 2019). Based on the diversity of PTMs in mammalian cells and the caveats described above, it is conceivable that several other α-synuclein PTMs still need to be uncovered by improved mass spectrometry techniques, such as top-down analysis (Breuker, 2018). We dedicated this review to discuss recent findings on α-synuclein SUMOylation and its possible implications in PD.

## The SUMOylation Pathway

Small Ubiquitin-like Modifier (SUMO) is a family of proteins that are covalently conjugated to lysine residues on a ΨKX(D/E) consensus motif of target proteins, where Ψ represents a hydrophobic residue, K is a target lysine, X is any residue, and D/E represent acidic residues (Matunis et al., 1996; Rodriguez et al., 2001; Zhao et al., 2014). SUMOylation regulates intra-nuclear cellular events, including cell division, transcription, DNA repair and nuclear transport (Zhao, 2018). Nevertheless, several studies have shown that extranuclear proteins can also be SUMOylated, implicating SUMOylation in several extranuclear roles, such as endocytosis, synaptic activity and signaling transduction (Henley et al., 2018). At the protein level, SUMOylation can affect the structure, stability, localization and protein-protein interaction. Moreover, several SUMOylation targets are proteins related to neurodegeneration, including tau and α-synuclein (Dorval and Fraser, 2006).

Protein SUMOylation involves sequential events that rely on the activation of three enzymes, known as E1 SUMO-activating, E2 SUMO-conjugating and E3 SUMO-ligase, each one uniquely contributes to the completion of this cascade and the final SUMOylation of the substrate (Muller et al., 2001). The SUMO peptide is first matured for conjugation by SUMO cysteine proteases that belong to the ubiquitin-like-specific protease 1 (Ulp)/sentrin-specific protease (SENP) family, which expose a glycine residue at its C-terminus (Hickey et al., 2012). The E1 SUMO-activating enzyme, a SAE1/2 heterodimer, then forms a thioester bond between its active-site cysteine and the mature SUMO peptide, in an ATP-dependent matter. The attached SUMO is subsequently transferred to a cysteine at the active-site of Ubc9, the only known E2 SUMO-conjugating enzyme of the pathway (Wilkinson and Henley, 2010). Finally, the E3 SUMO-ligase plays a facilitating role in the transfer of SUMO from Ubc9 to the substrate, allowing the completion of the substrate SUMOylation (Kahyo et al., 2001). It is important to note that different from the ubiquitin system, the E2 SUMO-conjugating enzyme Ubc9 can, to a certain extent, directly transfer SUMO to the substrate (Knipscheer et al., 2007). Recycling of SUMO peptides occurs as a result of deSUMOylation of SUMOylated proteins also by the SUMO proteases from the Ulp/SENP family.

Mechanistically, ubiquitination and SUMOylation share several common features. As ubiquitination, SUMOylation is a dynamic multistep conjugation and de-conjugation cascade, directed by ATP-dependent enzymes. Also, similar to ubiquitin, SUMO can be covalently attached to a single (monoSUMOylation) or multiple (multi-monoSUMOylation) lysine residues, or form SUMO chains (polySUMOylation) on the substrate protein (Wilkinson and Henley, 2010; Hickey et al., 2012). On the other hand, some features of the SUMO pathway are different from those of ubiquitination. For instance, while the ubiquitin system relies on a single ubiquitin isoform, the SUMOylation pathway exhibits several distinct SUMO isoforms, including the extensively studied SUMO1 to SUMO3, and the more recently described SUMO4 and SUMO5 isoforms (Bohren et al., 2004; Wilkinson and Henley, 2010). SUMO2 and SUMO3 share a high degree of similarity and are therefore regarded as SUMO2/3. Further distinguishing SUMO isoforms, SUMO2/3, but not SUMO1, support polySUMOylation with chain formation (Tatham et al., 2001). The additional SUMO isoforms, SUMO4 and SUMO5, were described to support polySUMOylation, but their roles await further investigation (Liang et al., 2016; Baczyk et al., 2017). Another critical difference is that ubiquitination usually serves as a signal for proteasomal protein degradation, while SUMOylation either affects lysine ubiquitination or regulates cellular activities, such as protein shuttling between compartments and activity of proteins (Matunis et al., 1996; Hershko and Ciechanover, 1998; Wilson, 2017).

SUMOylation machinery and protein SUMOylation dramatically increase in response to cellular stresses, including heat shock, DNA damage, viral infections, and nutrient and hypoxic stress, suggesting that SUMOylation could regulate the ability of cells to cope with stressors (Zhou et al., 2004; Enserink, 2015). It was suggested that SUMO2/3 was responsive to the different types of cellular stressors (Saitoh and Hinchey, 2000), while SUMO1 was regarded as a housekeeping conjugation involved in the regulation of protein subcellular localization and ubiquitination (Matunis et al., 1996). However, several studies support a broader scenario, where SUMO1 may also mediate the response to several cellular stressors, including heat-shock as well as genotoxic and hypoxic stress (Hong et al., 2001; Huang et al., 2003; Agbor et al., 2011). In agreement, proteasome inhibition and the proteotoxic stress it causes leads to the increased SUMOylation of proteins by SUMO2/3 or SUMO1, which accumulate in the promyelocytic leukemia protein (PML) nuclear bodies (Sha et al., 2019). Supporting an even broader role of SUMO isoforms in response to stress, polymorphism in the SUMO4 gene was linked to type 1 diabetes mellitus and increased SUMOylation by SUMO4 was detected in pre-eclamptic placentas (Bohren et al., 2004; Baczyk et al., 2017).

## SUMOylation in PD

The occurrence of PD is associated with exposure to several types of stressors, including toxins and viral infections, as well as inflammation. Populations exposed to insecticides, such as paraquat and rotenone, have increased incidence of PD (Tanner et al., 2011). Also, parkinsonism can occur after influenza viral infections followed by encephalitis lethargica (Maurizi, 1987), and studies injecting the influenza H5N1 strain in mice caused overt neuroinflammation, accumulation of α-synuclein and nigral degeneration (Jang et al., 2009). Brain trauma and the neuroinflammation it produces, also lead to the accumulation of α-synuclein and increase the risk of developing PD (Newell et al., 1999). Because these insults activate the SUMO machinery (Enserink, 2015), SUMOylation may mediate the toxic outcomes of the different stressors in PD and other α-synucleinopathies. In agreement, injection of rotenone in mice brain leads to a concomitant increase of higher molecular weight species of SUMO1 and α-synuclein (Weetman et al., 2013). Moreover, the treatment of oligodendroglial cells with polyunsaturated fatty acids followed by oxidative stress lead to the formation of α-synuclein aggregates positive for ubiquitin and SUMO1 (Riedel et al., 2011).

SUMOylation participates in several pathways connected to PD, including changes in α-synuclein biology and pathology (discussed in detail in the sections below), regulation of DJ-1 activity (Ariga et al., 2013), modulation of transcription factors involved in mitochondrial and lysosomal biogenesis (Harder et al., 2004), and regulation of mitochondrial fission machinery (Harder et al., 2004; Guo et al., 2013).

Mutations in the DJ-1 gene lead to autosomal recessive early-onset parkinsonism (Bonifati et al., 2003). DJ-1 is present in different cellular compartments and has several attributed functions, among them transcriptional regulation, antioxidative properties, chaperone and mitochondrial regulation (Ariga et al., 2013). In particular, the DJ-1 redox-dependent molecular chaperone activity was suggested to inhibit α-synuclein aggregate formation (Shendelman et al., 2004). DJ-1 was also shown to directly bind the mitochondrial F_0_F_1_-ATP synthase b subunit enabling proper mitochondrial function, a process that was prevented by DJ-1 PD mutations (Chen et al., 2019), highlighting the importance of DJ-1 to maintain mitochondrial and α-synuclein homeostasis. SUMOylation of DJ-1 was shown to be essential to enable DJ-1 transcriptional activity, and the consequent neuroprotection it exerts, a function that is prevented by PD mutations (Shinbo et al., 2006). Future investigation of the levels of DJ-1 SUMOylation in PD may further shed light on the role of SUMOylation in the disease.

Accumulation of dysfunctional mitochondria is observed in affected PD brain regions, in particular in the proximity or even inside Lewy bodies (Shahmoradian et al., 2019). Also, mitochondrial complex I activity is consistently decreased in dopaminergic neurons of the substantia nigra (Schapira et al., 1989). Moreover, genome-wide expression studies identified that bioenergetics-related nuclear genes controlled by the mitochondrial biogenesis master regulator, PGC-1a, are underexpressed in PD patients (Zheng et al., 2010), suggesting that PGC-1a activity may be decreased in PD and may contribute to the mitochondrial dysfunction observed in the disease. In this framework, SUMOylation of PGC-1a was shown to reduce its transcriptional activity in HeLa cells (Rytinki and Palvimo, 2009), raising the possibility that increased SUMOylation could play a role in the accumulation of dysfunctional mitochondria in the disease.

Another aspect critical for the proper mitochondrial function is the dynamics of the organelle, dictated by fusion and fission processes (Cao et al., 2017). PINK1 and parkin, are genes mutated in autosomal recessive PD that regulate mitochondrial dynamics. More specifically, the PINK1/parkin pathway regulates mitochondrial dynamics by promoting mitochondrial fission as their PD mutants reduce mitochondrial fission (Poole et al., 2008). The dynamin-related protein 1 (DRP-1) is a critical GTPase that promotes mitochondrial fission by encircling constricted mitochondria and assisting the formation of two separated mitochondria (Youle and Karbowski, 2005). DRP-1 was shown to be SUMOylated by SUMO1 and SUMO2/3 isoforms (Harder et al., 2004; Figueroa-Romero et al., 2009), and a surprising number of SUMO1 conjugates were found in mitochondria at fission sites of COS-7 cells, implying that SUMOylated DRP-1 could facilitate the fragmentation of the organelle (Harder et al., 2004). On the other hand, DRP-1 conjugation to SUMO2/3 was shown to prevent mitochondrial fragmentation and to protect against neuronal damage after oxygen/glucose deprivation (Guo et al., 2013). Although the definitive role of SUMOylation on DRP-1 ability to promote mitochondrial fission requires further clarification, the differences in the experimental models (COS-7 cells *vs.* primary neurons) and the different SUMO isoforms analyzed (SUMO1 *vs.* SUMO2/3) may help explain the differences. Additional studies addressing the role of DRP-1 SUMOylation in PD may help understand the mechanisms involved in the accumulation of dysfunctional mitochondria in the disease and their connection to the increased aggregation of α-synuclein (Lautenschager and Kaminski Schierle, 2019).

Other proteins involved on α-synuclein homeostasis have also been connected to the SUMO pathway. Lysosomal function is suggested to be dysfunctional in PD (Dehay et al., 2013), and SUMOylation of the Transcription Factor EB (TFEB), a master regulator of lysosomal biogenesis, decreases its transcriptional activity (Miller et al., 2005). Thus, increased TFEB SUMOylation could hamper the renewal of lysosomes to properly carry out α-synuclein degradation through the lysosomal-autophagy pathway.

Another protein implicated in PD is HSP90, which interacts with α-synuclein and is also present in Lewy bodies (Uryu et al., 2006). The role of HSP90 in α-synuclein aggregation is still a matter of debate. Some studies suggest that HSP90 inhibits α-synuclein aggregation (Daturpalli et al., 2013), while inhibition of HSP90 was also shown to prevent α-synuclein oligomer formation and to rescue α-synuclein-induced toxicity (Putcha et al., 2010). Although HSP90 actions in PD are not fully understood, chemical SUMOylation of HSP90 was recently shown to modulate co-chaperone binding *in vitro* (Wolmarans et al., 2019). Therefore, SUMOylation may play a role in the ability of HSP90 to modulate α-synuclein aggregation into Lewy bodies.

SUMO1 and one of α-synuclein SUMO-ligase, PIAS2, were detected in Lewy bodies of PD brains (Kim et al., 2011; Rott et al., 2017). Most importantly, the levels of SUMOylated α-synuclein isolated by immunoprecipitation are increased in the cerebral cortex of PD patients with dementia (Rott et al., 2017). Moreover, SUMO1 is also associated with lysosomal remnants in glial cytoplasmic inclusions of Multiple System Atrophy (MSA) brains (Pountney et al., 2005; Wong et al., 2013; Radford et al., 2014), and SUMO1 is found by confocal microscopy to co-localize with both α-synuclein and HSP90 in the disease inclusions (Wong et al., 2013). While data of immunoprecipitation experiments suggest that HSP90 may be conjugated to SUMO1 in glial cytoplasmic inclusions (Wong et al., 2013), the validation of SUMO1 conjugation to α-synuclein in MSA brains still need further investigation. Overall, although SUMO1 covalent conjugation to α-synuclein in α-synucleinopathy lesions need to be confirmed by improved mass spectrometry techniques, the current findings suggest that different proteins present in α-synucleinopathy lesions, including α-synuclein (Rott et al., 2017) and HSP90 (Uryu et al., 2006), could be SUMOylated and could also play a role in neurodegeneration. Altogether, understanding the regulation of α-synuclein SUMOylation, and possibly other proteins (such as HSP90), may help shed light on the mechanisms that lead to PD and other α-synucleinopathies.

## α-Synuclein SUMOylation

α-synuclein contains two SUMOylation consensus lysines (Lys96 and Lys102), which favor conjugation by SUMO1 over SUMO2/3 (Dorval and Fraser, 2006; Table 2). Different SUMO-ligases SUMOylate α-synuclein, including human Polycomb protein 2 (hPc2), PIAS2 and TRIM28 (Krumova et al., 2011; Oh et al., 2011; Rott et al., 2017; Rousseaux et al., 2018), and they tend to lead to predominant monoSUMOylation or multi-monoSUMOylation (Krumova et al., 2011; Rott et al., 2017). Specific α-synuclein SUMOylation by the SUMO-ligases was confirmed using different experimental approaches, including *in vitro* conjugation assays and immunoprecipitations using cell lines, primary neuronal cultures as well as brain tissues (Krumova et al., 2011; Rott et al., 2017; Rousseaux et al., 2018; Zhu et al., 2018).

**TABLE 2:** α-synuclein SUMOylation.

SUMO isoform	Conjugation type of α-synuclein	E3 SUMO ligase	Functional consequences of α-synuclein SUMOylation
SUMO1	monoSUMOylation	None added	SUMO1 conjugation to α-synuclein favored over SUMO2/3 in HEK293 cells (Dorval and Fraser, 2006)
		
		hPc2	Promotes the aggregation of α-synuclein and reduces staurosporine-induced cell death in COS-7 cells (Oh et al., 2011)
		
		PIAS2	Decreases α-synuclein ubiquitination and proteasomal degradation, increasing its levels of in HEK293 cells (Rott et al., 2017) Promotes the aggregation of α-synuclein in SH-SY5Y cells (Rott et al., 2017)
	
	monoSUMOylation in *E. coli*	None added	Inhibition of α-synuclein fibrillation *in vitro* (Krumova et al., 2011)
	
	Semisynthetic monoSUMOylation	None added	Inhibition of α-synuclein fibrillation *in vitro* (Abeywardana and Pratt, 2015)

SUMO2/3	polySUMOylation	Trim28	Trim 28 knockout decreases α-synuclein SUMOylation and its levels (Rousseaux et al., 2018)
		
		None added	Promotes the nuclear translocation of α-synuclein in SH-SY5Y cells (Ryu et al., 2019)
	
	SUMO2 fusion to C-terminus of α-synuclein	None added	Mutation of α-synuclein lysines 96 and 102 prevents its extracellular vesicle sorting (Kunadt et al., 2015)
	
	Semisynthetic monoSUMOylation	None added	Inhibition of α-synuclein fibrillation *in vitro* (Abeywardana and Pratt, 2015)

SMT3 (yeast)	monoSUMOylation	None added	Impairment of SUMOylation increases α-synuclein toxicity and foci formation in *S. cerevisiae* (Shahpasandzadeh et al., 2014) Increase in α-synuclein Ser129 phosphorylation counteracts α-synuclein toxicity and foci formation caused by inhibition of its SUMOylation (Shahpasandzadeh et al., 2014)

Undetermined SUMO isoform			Mutation of α-synuclein lysines 96 and 102 increase its aggregation and toxicity in HEK293 cells and a PD rat model (Krumova et al., 2011)

According to the consensus motif (Ψ-K-x-D/E), α-synuclein lysines 96 and 102 have a very high SUMOylation score (Zhao et al., 2014). Nevertheless, mass spectrometry analysis of purified α-synuclein from SUMO2 transgenic mice identified that several other α-synuclein lysines (11 out of the total 15) are SUMOylated, implying that α-synuclein is also SUMOylated in non-consensus lysines *in vivo* (Krumova et al., 2011). Accordingly, mutations in Lys96 and Lys102 promoted only a partial decrease in α-synuclein SUMOylation (Krumova et al., 2011; Rott et al., 2017). Altogether, the variety of α-synuclein lysine residues that can be conjugated by SUMO, together with the redundancy of SUMO-ligases and SUMO isoforms that can contribute to α-synuclein SUMOylation, support the idea that differences in SUMOylation patterns could lead to a complex regulation of α-synuclein normal and pathological functions, including degradation, intracellular distribution, protein interactions and aggregation.

## SUMOylation Affecting the Subcellular Localization of α-Synuclein

**FIGURE 1:**
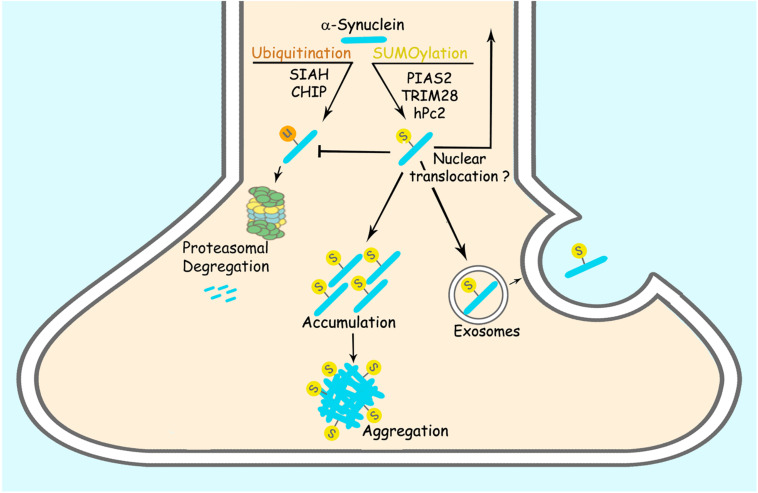
SUMOylation regulates α-synuclein accumulation and aggregation. PIAS2, TRIM28 and hPc2 SUMOylate α-synuclein. α-synuclein SUMOylation counteracts its ubiquitination and proteasomal degradation, leading to increased α-synuclein steady-state levels. SUMOylation of α-synuclein also leads to its increased aggregation *per se*. SUMOylation of α-synuclein was also suggested to increase its exosomal release and nuclear translocation.

SUMOylation is involved in targeting proteins to different subcellular compartments, in particular to the nucleus (Pichler and Melchior, 2002). Thus, it is conceivable that SUMOylation could play a critical role in the intracellular targeting of α-synuclein. Even though α-synuclein is primarily located at nerve terminals; recent biochemical analysis has consistently identified α-synuclein in the nucleus of neurons (Pinho et al., 2019). Aggregated and Ser129 phosphorylated α-synuclein accumulate in the nucleus of neurons of DLB brains (Pinho et al., 2019). Additional studies have reported that α-synuclein is present in the nucleus of nigral neurons of mice upon injection of paraquat (Goers et al., 2003). Besides, α-synuclein interacts and aggregates with histones (Goers et al., 2003; Kontopoulos et al., 2006; Jiang et al., 2017). Supporting the possible role of SUMOylation in the regulation of α-synuclein translocation to the nucleus, the nuclear SUMO-ligase TRIM28 promotes the accumulation of α-synuclein in the nucleus of neurons (Rousseaux et al., 2016; Figure 1). Another study showed increased nuclear translocation of SUMOylated α-synuclein upon interaction with karyopherin (Ryu et al., 2019).

A recent report failed to observe significant SUMOylation of extranuclear proteins using a His_6_-HA-SUMO1 knock-in mouse model (Daniel et al., 2017). Nevertheless, because this knock-in mouse model has an approximate 30% decreased of Flag-HA-SUMO1 expression (Daniel et al., 2017), it may hinder the detection of protein SUMOylation that occurs outside the nucleus, such as synaptic and mitochondrial proteins (Henley et al., 2018). In this framework, SUMOylation of α-synuclein was also suggested to influence its release from neurons. SUMOylation-deficient α-synuclein was shown to have decreased extracellular release, implying that SUMOylation of α-synuclein may increase its binding to membranes, facilitating, therefore, its release from cells within extracellular vesicles (Kunadt et al., 2015; Figure 1 and Table 2). In addition, the study showed that SUMO2 *per se* is also targeted to extracellular vesicles, in a process requiring the Endosomal Sorting Complex Required for Transport (ESCRT) complex (Kunadt et al., 2015), suggesting that ESCRT machinery could also be a SUMOylation target and as a consequence, could indirectly regulate cell-to-cell propagation of α-synuclein aggregates or seeds.

Moreover, further supporting a more widespread role of SUMOylation, a recent super-resolution microscopy study found that, although SUMO1, SUMO2/3 and Ubc9 are mainly nuclear in neurons, they also partially co-localize with pre- and post-synaptic markers, such as synaptophysin and PSD95 (Colnaghi et al., 2019). Since α-synuclein is a pre-synaptic protein, the finding of SUMOylation machinery at the pre-synapse further support the importance of α-synuclein SUMOylation. In this framework, it is possible that SUMOylation may also modulate the role of α-synuclein at the presynapse.

## The Effect of SUMOylation on α-Synuclein Levels

SUMOylation regulates the stability of a plethora of proteins (Galisson et al., 2011), implying that the degree of SUMOylation could also modulate the levels of α-synuclein in the brain. While SUMOylation can promote the degradation of proteins by priming SUMO-targeted ubiquitin ligases (STUbLs) (Uzunova et al., 2007), SUMOylation can also outcompete and prevent the ubiquitination and proteasomal degradation of different proteins (Desterro et al., 1998; Rott et al., 2017; Table 2). SUMOylation of α-synuclein by PIAS2 and TRIM28 promotes its accumulation (Rott et al., 2017; Rousseaux et al., 2018) while hPc2 causes no change in α-synuclein levels (Oh et al., 2011), indicating that different SUMO-ligases may promote distinct forms of α-synuclein SUMOylation, where some of them would prevent α-synuclein degradation while others would not. In this framework, knockdown of PIAS2 and TRIM28 in HEK293 cells and neurons resulted in decreased levels of α-synuclein that were not ascribed to changes in expression (Rousseaux et al., 2016; Rott et al., 2017). Accordingly, SUMOylation of α-synuclein by PIAS2 competes with and decreases the α-synuclein ubiquitination promoted by both the ubiquitin-ligases SIAH-2 and Nedd4 (Rott et al., 2017; Figure 1). In agreement, PIAS2 knockdown increases SIAH-2-dependent α-synuclein monoubiquitination and proteasomal degradation (Rott et al., 2017). Although TRIM28 knockout mice show decreased levels of α-synuclein, the mechanism by which TRIM28 leads to the accumulation of α-synuclein is still not clear (Rousseaux et al., 2018). Further supporting the role of SUMOylation in counteracting α-synuclein ubiquitination and degradation, pharmacological treatment with the SUMO E1 inhibitor, ginkgolic acid, decreases the steady-state levels of α-synuclein in both HEK293 cells and primary neuronal cultures by promoting its degradation through the proteasome (Rott et al., 2017). In an experimental condition where α-synuclein aggregates were formed by depolarizing SH-SY5Y cells and neurons with KCl, inhibition of SUMOylation by ginkgolic acid stimulated the clearance of α-synuclein aggregates by promoting macroautophagy (Vijayakumaran et al., 2019). Therefore, ginkgolic acid could represent a viable therapeutic strategy to increase proteasomal degradation of ubiquitinated α-synuclein as well as α-synuclein aggregates by macroautophagy stimulation.

SUMOylation also increases the levels of tau, huntingtin, ataxin-3 and SOD1 (Fei et al., 2006; O’Rourke et al., 2013; Zhou et al., 2013; Luo et al., 2014; Ochaba et al., 2016), supporting a broader role of SUMOylation in neurodegenerative diseases. Therefore, dysregulation of SUMOylation may lead to the accumulation of critical proteins involved not only in PD and other α-synucleinopathies but also in additional neurodegenerative diseases, such as Alzheimer’s disease, Huntington’s disease, Spinocerebellar Ataxia-3 and amyotrophic lateral sclerosis.

## SUMOylation and α-Synuclein Aggregation

The synthetic fusion of yeast SUMO, encoded by the SMT3 gene, as well as proteins such as maltose-binding protein and NusA, to the N- or C-terminus of recombinant proteins, increase the folding and solubilization of proteins *in vitro* (Zuo et al., 2005; Marblestone, 2006). Nevertheless, studies using approaches to increase SUMOylation of internal lysine residues of proteins in cell lines and in *in vivo* models have shown the opposite effect. For instance, SUMOylation of neurodegenerative-related proteins, including tau, huntingtin, SOD1 and α-synuclein, have shown to increase their aggregation (Fei et al., 2006; O’Rourke et al., 2013; Luo et al., 2014; Ochaba et al., 2016; Rott et al., 2017). In addition to differences in the attachment of SUMO isoforms to target proteins (whether to N- or C-terminus or to internal lysines), variations in the SUMO isoforms themselves may play a role in changing the solubility of proteins. For instance, yeast SUMO may have different aggregation properties compared to human SUMO1 and SUMO2/3. Along with this possibility, yeast SUMO shares 47% homology with SUMO1, which in turn is 50% homologous to SUMO2/3 (Melchior, 2000; Marblestone, 2006). In this framework, α-synuclein can be conjugated to either SUMO1 or SUMO2/3, which give rise to mono- or polySUMOylation, respectively (Table 2).

How could we explain the ability of conjugated SUMO to increase the aggregation of disease-causing proteins? One possibility is that proteins involved in neurodegenerative diseases are intrinsically prone to aggregation and that different PTMs with some hydrophobic character would more easily tilt the equilibrium toward their aggregation (O’Rourke et al., 2013; Morimoto et al., 2016). Another possibility is that excessive SUMOylation at multiple lysine residues, due to enhanced SUMOylation machinery upon aging and stress (Tempe et al., 2008; Scurr et al., 2017; Henley et al., 2018), could trigger the aggregation of crucial proteins in different neurodegenerative diseases. Alternatively, non-covalent interaction between SUMOylated disease-causing proteins and adjacent SUMOylated proteins, could work as a molecular “glue” (Matunis et al., 2006), facilitating the formation of aggregates in neurodegenerative diseases.

Regarding α-synuclein, SUMOylation by PIAS2 promotes its aggregation both *in vitro* and in cell lines (Rott et al., 2017). This aggregation was observed without any additional treatment to the *in vitro* reactions and cells (Rott et al., 2017), supporting the notion that SUMOylation by PIAS2 *per se* is enough to promote α-synuclein aggregation (Figure 1). In agreement, α-synuclein PD mutants (A53T, A30P, and E46K) are significantly more SUMOylated than the wild type protein, leading to their robust aggregation (Rott et al., 2017). Accordingly, substantia nigra of PD patients contains increased formic acid- and proteinase K-resistant SUMOylated proteins, as well as SUMOylated α-synuclein (Rott et al., 2017). Stress caused by the accumulation of α-synuclein inclusions may further contribute to the increase of α-synuclein SUMOylation in a positive-feedback loop, as suggested by the formation of SUMO positive aggresome-like α-synuclein inclusions in different cell lines treated with proteasome inhibitors (Kim et al., 2011; Oh et al., 2011; Wong et al., 2013).

On the other hand, in a paradigm where a PDZ binding domain-tagged α-synuclein was co-expressed with PDZ domain-EGFP in HEK293 cells, the aggregation of EGFP (used as a marker for α-synuclein aggregation) increased when lysines 96 and 102 were mutated to arginines (Krumova et al., 2011), suggesting that SUMOylation would decrease the aggregation of α-synuclein. The reason for the discrepancies in the possible role of SUMOylation on α-synuclein aggregation in cell lines is not clear. However, they could be related to differences in the α-synuclein constructs used. For instance, the interaction of α-synuclein with EGFP could facilitate their aggregation, making them more noticeable even in the absence of any method to remove non-aggregated α-synuclein, such as detergent or proteinase K (Rott et al., 2008, 2017). Alternatively, differences of SUMO isoforms and SUMO-ligases could also contribute to the apparent discrepancies of α-synuclein aggregation seen in the different studies. Accordingly, in yeast, impaired SUMOylation by SMT3 results in increased aggregation of α-synuclein (Shahpasandzadeh et al., 2014; Table 2), suggesting that not only the SUMO isoform but also the protein context may also play a role in the aggregation of SUMOylated α-synuclein.

The role of SUMOylation in the formation of α-synuclein fibrils was also investigated. *In vitro* SUMOylation, either by conjugation or by a semi-synthetic approach, reduce the ability of α-synuclein to form fibrils (Krumova et al., 2011; Abeywardana and Pratt, 2015). The reduction in fibrillation was more pronounced when SUMO was attached to Lys102 compared to when it was attached to Lys96 (Abeywardana and Pratt, 2015). Moreover, SUMO1 was more efficient in reducing fibrillation than SUMO3 (Abeywardana and Pratt, 2015), further supporting the possibility of SUMO isoform-dependent α-synuclein aggregation and fibrillation. Although these findings suggest that SUMOylation may hinder the formation of α-synuclein fibrils, SUMOylation still supports the formation of oligomeric α-synuclein aggregates (Rott et al., 2017), suggesting that SUMOylation could inhibit the conversion of α-synuclein oligomeric into fibrils forms. Because oligomeric α-synuclein has been suggested to be toxic to neurons and non-fibrillar proteinaceous material are prominent at the core of Lewy bodies (Winner et al., 2011; Fusco et al., 2017), inhibition of the transition from oligomeric to fibril forms by SUMOylation could help explain the contribution of SUMO to α-synuclein toxicity in PD.

## SUMOylation of α-Synuclein and Toxicity

The aggregation of α-synuclein increases upon conjugation with SUMO1 and α-synuclein PD mutants have increased tendency to be SUMOylated, triggering their prompt and robust aggregation (Rott et al., 2017), supporting the idea that SUMOylation may mediate the accumulation of toxic α-synuclein species. Also, knockdown of TRIM28 increased dopaminergic neuron survival in a transgenic mice model overexpressing α-synuclein (Rousseaux et al., 2016).

On the other hand, α-synuclein SUMOylation by hPc2 increases COS-7 cell survival after exposure to the apoptosis inducer staurosporine (Oh et al., 2011). Also, expression of α-synuclein K96R, K102R mutants in cultured neurons, and rat substantia nigra displayed increased toxicity (Krumova et al., 2011), suggesting that SUMOylation of α-synuclein could also play a protective role depending on the cellular context and SUMO-ligase employed. Nevertheless, because lysine residues are conjugated to several PTMs in addition to SUMOylation, including ubiquitination and glycation (Zee and Garcia, 2012), the use of α-synuclein constructs with mutated lysines are challenging to interpret due to the possible confounding effects with different PTMs. Overall, a better understanding of how SUMOylation may contribute to α-synuclein toxicity will provide essential clues to the mechanisms of neuronal toxicity observed in the disease.

## The Interplay Between α-Synuclein SUMOylation and Phosphorylation

In a recent proteomic study, almost ten percent of protein SUMOylation occurred proximal to phosphorylation sites, and several SUMOylation sites were dependent on prior phosphorylation events, implying a significant cross-talk between SUMOylation and phosphorylation (Hendriks et al., 2017). Since phosphorylation at Ser129 is an abundant PTM in Lewy bodies (Fujiwara et al., 2002; Anderson et al., 2006), an interplay between SUMOylation and phosphorylation may also occur in α-synuclein. Supporting this possibility, SUMOylation of α-synuclein in yeast down-regulates Ser129 phosphorylation promoted by CDK5 and PLK2 and increases the clearance of α-synuclein aggregates (Shahpasandzadeh et al., 2014). There are still no studies investigating whether α-synuclein SUMOylation may also influence Ser129 phosphorylation of α-synuclein in mammalian cells. Nevertheless, in an example of their interplay in mammalian cell models relevant to neurodegenerative diseases, SUMOylation of tau induces its hyperphosphorylation at multiple AD-related sites (Luo et al., 2014). In a reciprocating manner, phosphorylation of tau increases its SUMOylation, leading to tau accumulation and aggregation (Luo et al., 2014), suggesting that in a more complex *milieu*, such as mammalian cells, SUMOylation and phosphorylation could lead to a feed-forward mechanism to increase protein aggregation and pathology. Future studies need to be carried out to investigate if and how α-synuclein SUMOylation and phosphorylation influence each in neuronal models.

## Concluding Remarks

Based on previous findings that proteins SUMOylation increases upon stress and that several disease-causing proteins aggregate and become toxic upon SUMOylation, we hypothesize that, among the *repertoire* of α-synuclein modifications, SUMOylation may represent a PTM that can lead to α-synuclein pathology. Supporting this possibility, several studies have shown that α-synuclein is SUMOylated in different cell models and brain tissues, and that SUMOylation by certain SUMO-ligases and SUMO isoforms leads to α-synuclein accumulation, aggregation and toxicity. Most importantly, SUMOylated α-synuclein is increased in α-synucleinopathy brains. In conclusion, even though studies using improved mass spectrometry techniques are required to allow a more detailed characterization of SUMOylated α-synuclein in brain tissues, the biochemical and cell biological findings indicate that α-synuclein SUMOylation may represent a novel target for better therapeutic intervention.

## Author Contributions

Both authors listed have made a substantial, direct and intellectual contribution to the work, and approved it for publication.

## Conflict of Interest

The authors declare that the research was conducted in the absence of any commercial or financial relationships that could be construed as a potential conflict of interest.

## Abbreviations

PD: Parkinson’s disease
SIAH: Seven *in Absentia* homolog.
